# Preoperative Plasma Fibrinogen Level as a Significant Prognostic Factor in Patients With Localized Renal Cell Carcinoma After Surgical Treatment

**DOI:** 10.1097/MD.0000000000002626

**Published:** 2016-01-29

**Authors:** Hakmin Lee, Sang Eun Lee, Seok-Soo Byun, Hyeon Hoe Kim, Cheol Kwak, Sung Kyu Hong

**Affiliations:** From the Department of Urology, Seoul National University Bundang Hospital, Seongnam (HL, SEL, S-SB, SKH); and Department of Urology, Seoul National University Hospital, Seoul, Korea (HHK, CK).

## Abstract

We sought to investigate the association of preoperative fibrinogen levels with clinicopathologic outcomes after surgical treatment of nonmetastatic renal cell carcinoma. We reviewed the records of 1511 patients who had their fibrinogen levels measured preceding surgery. The associations between preoperative fibrinogen level and risk of adverse clinicopathologic outcomes were tested using the multivariate logistic regression and multiple Cox-proportional hazards model, respectively.

Based on plasma fibrinogen levels, we stratified the patients into 2 groups with a cut-off value of 328 mg/dL. Kaplan–Meier analysis showed significantly inferior survival outcomes in progression-free (*P* < 0.001), cancer-specific (*P* < 0.001), and overall survival (*P* < 0.001). In multivariate analyses, a high fibrinogen level (≥328 mg/dL) was significantly related to a higher Fuhrman grade (hazard ratio [HR] 1.374, *P* = 0.006) and a larger tumor size (≥7 cm) (HR 2.364, *P* < 0.001). Multivariate Cox analysis also revealed that a high preoperative fibrinogen level is a significant predictor for poor disease progression (HR 1.857, *P* < 0.001), cancer-specific survival (HR 3.608, *P* = 0.003), and overall survival (HR 1.647, *P* = 0.027).

Increased plasma fibrinogen levels were significantly associated with poor pathological features and worse survival outcomes in patients with nonmetastatic renal cell carcinoma after surgical treatment. Further evaluations such as prospective randomized trials are needed to understand the underlying mechanism for these associations.

## INTRODUCTION

Renal cell carcinoma (RCC) is the most frequently diagnosed malignant tumor of the kidneys.^1^ According to the developments of modern imaging tools, the incidence of RCC is also steadily increased during last decades.^2^ Moreover, the incidental diagnosis of small renal mass became more frequent. Although some studies showed noninferior survival outcomes for nonsurgical treatments for small renal masses,^3,4^ the gold standard for curative treatment for those small renal tumors is surgical resection.^5^ Therefore, it is becoming more important to identify the prognostic factors that can predict the patients’ clinical outcomes in those localized RCC patients who underwent surgical treatment.

It is well known that cancer patients present with various abnormalities of hemostatic laboratory biomarkers, particularly in coagulation markers.^6^ Some epidemiologic studies reported that there is a 4- to 7-fold increased risk of venous thromboembolism in patients with malignancy compared with the normal population.^7,8^ However, the exact pathogenesis of cancer-related coagulopathy is still not fully understood and seems to be multifactorial. Moreover, the coagulation system has been suggested to be closely related to the aggressive behavior of cancer or its metastatic capacity.^9,10^ Recently, several clinical studies showed a significant relationship between high plasma fibrinogen levels and clinic-pathologic outcomes in various cancer types, such as endometrial, ovarian, colorectal, and upper tract urinary cancer.^11–14^ Most of these studies demonstrated that the increased plasma fibrinogen levels are closely related to increased rate of metastasis and impaired survival outcomes after the treatment of corresponding malignancies. However, there has been lack of attention to these relationships in RCC to date.

In the present study, we tried to clarify the relationship between preoperative plasma fibrinogen levels and postoperative survival in our relatively large cohort consisting of patients with localized RCC who underwent surgical treatment from 2 tertiary institutions.

## METHODS

After approval from institutional review board (B-1510-318-117), we retrospectively reviewed records of 2310 patients who underwent surgical treatment for RCC from January 2006 to June 2013 in 2 tertiary centers. Among all subjects, 1679 patients who had fibrinogen measurements within 6 months before surgery were identified. Fibrinogen tests were included in the coagulation panel among the preoperative work-ups. But 631 patients did not have fibrinogen measurements because these patients had prothrombin time/activated partial thromboplastin time measurements instead of the coagulation panel and were excluded from the study. After additional exclusion of 168 patients (other malignancy [n = 7], metastatic disease [n = 129], bilateral synchronous disease [n = 12], and Von hippel Lindau disease [n = 11], surgical margin positive [n = 9]), 1511 subjects were finally analyzed. Patients’ clinicopathologic characteristics were acquired from prospectively managed database of both institutions. Abdominal computed tomography (CT), chest radiography (or CT), and bone scintigraphy were routinely included in preoperative radiologic evaluations. Histologic subtyping and pathologic staging were performed according to the 7th TNM classification of the Union for International Cancer Control and the American Joint Committee on Cancer guidelines,^15^ and cellular grading was performed using Fuhrman grading system.^16^ Disease progression was defined as local recurrence, distant metastasis, or mortality from RCC. Postoperative follow-ups were performed at 3 to 6-month intervals for the initial 2 years and yearly thereafter. Cancer-specific and overall mortalities were assessed using the Korea National Statistical Office database and/or by review of medical records. Progression-free survival, overall survival, and cancer-specific survival were assessed from the date of surgery to date of progression, cancer-specific mortality, and any-cause mortality on last follow-up, respectively.

*χ*^2^ tests and independent *t* tests were performed to evaluate the differences between subgroups. Logistic regression tests were used for univariate/multivariate analyses for adverse pathologic events. Kaplan–Meier analyses using the log-rank test were performed for the evaluation of survival outcomes according to the fibrinogen level. Multivariate Cox-proportional hazard analyses were used to identify the significant predictors for survival outcomes after surgical treatment. All statistical analyses were performed by SPSS software package (SPSS 19.0, Chicago, IL). All *P* values were 2-sided and values <0.05 were considered statistically significant.

## RESULTS

Clinical and pathologic characteristics of the patients are summarized in Table 1. Among all patients, the median age was 58 years (interquartile range [IQR] 49–67), and the median fibrinogen level was 332 mg/dL (IQR 279–410). The median follow-up time was 36 months (IQR 24–57) for all patients. To determine the optimal cut-off level for fibrinogen level, the receiver operating characteristic (ROC) curve of fibrinogen for cancer-specific mortality was analyzed. The area under the ROC curve of fibrinogen for cancer-specific mortality was 0.735. As the fibrinogen level of 328 mg/dL showed a maximal Youden's index on the ROC curve, we determined this value as a cut-off level to compare high/low fibrinogen level groups. Therefore, there were 787 patients in the high fibrinogen group (≥328 mg/dL) and 724 patients in the low fibrinogen group (<328 mg/dL). When we compared the clinical characteristics of the 2 groups, patients with high fibrinogen levels were significantly older (*P* < 0.001), had more comorbidities (hypertension, diabetes mellitus; both *P* < 0.001), and had larger tumor volumes (*P* < 0.001) than patients with low fibrinogen levels. In addition, the high fibrinogen group also showed worse pathological outcomes than the low fibrinogen group. Pathologic stage (*P* < 0.001) and cellular grade (*P* < 0.001) were significantly higher and adverse pathologic findings such as tumor necrosis (*P* = 0.013) and sarcomatoid differentiation (*P* = 0.003) were significantly more prevalent in the high fibrinogen group.

**TABLE 1:**
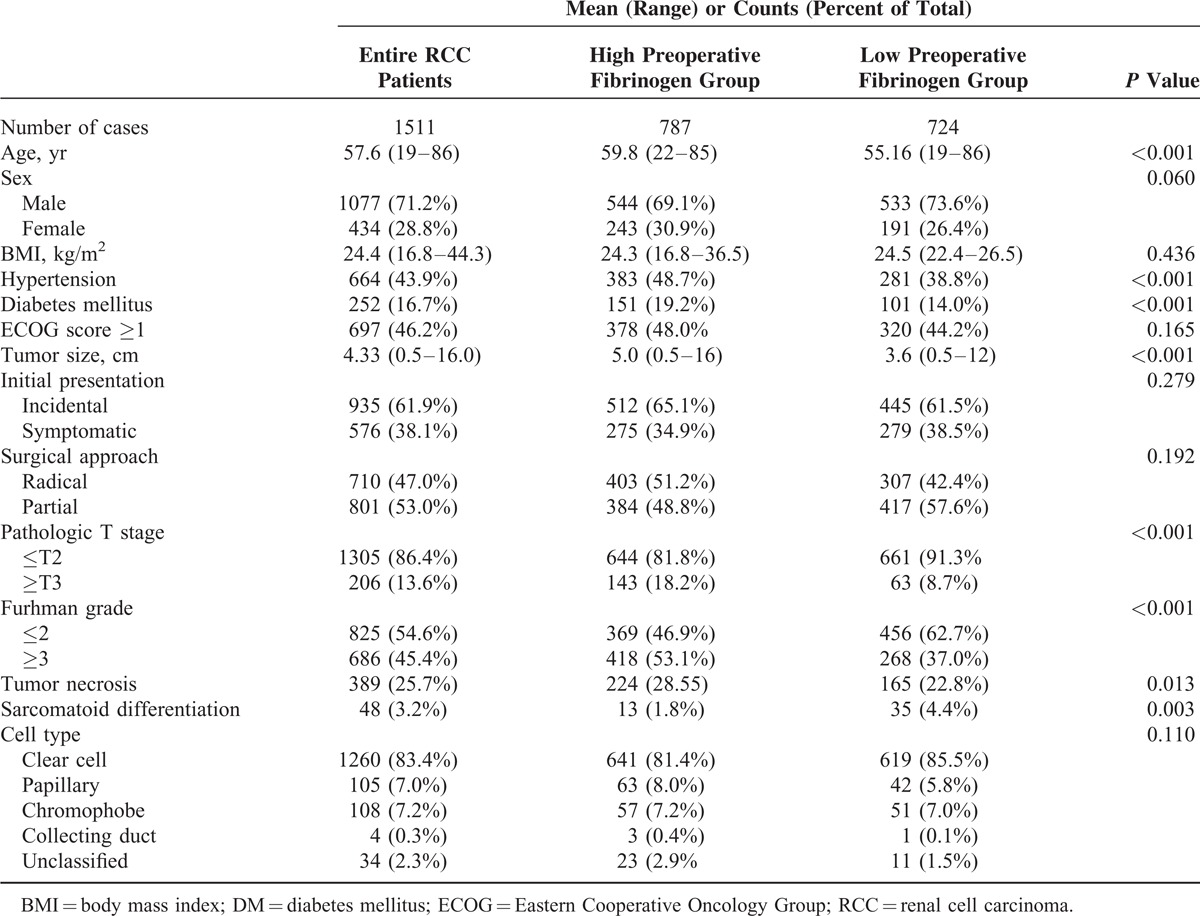
Clinical and Pathologic Characteristics of Entire RCC Patients and Subgroups by Fibrinogen Level

Multivariate logistic regression analyses were performed to evaluate whether fibrinogen level was related to several adverse pathologic outcomes (Table 2). Higher fibrinogen levels were significantly associated with several adverse pathologic outcomes, including higher cellular grade (≥3, *P* < 0.001), high pathologic stage (≥3, *P* < 0.001), larger tumor volume (≥7 cm, *P* < 0.001), tumor necrosis (*P* = 0.012), and sarcomatoid differentiation (*P* = 0.005) in univariate analyses. However, higher cellular grade (≥3; hazard ratio [HR] 1.374; 95% confidence interval [CI] 1.094–1.725; *P* = 0.006) and larger tumor size (≥7 cm; HR 2.364; 95% CI 1.707–3.273; *P* < 0.001) reached statistical significance in multivariate analysis when they were adjusted for age, body mass index, T stage, Eastern Cooperative Oncology Group (ECOG) score, and with/without cellular grade and tumor size.

**TABLE 2:**
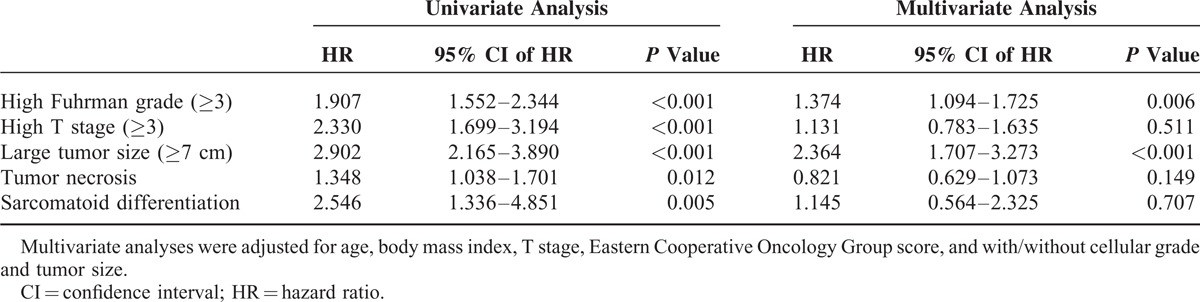
Multivariate Analyses of the Impact of High Fibrinogen Level (≥ 328 mg/dL) on Various Pathologic Outcomes After Surgical Treatment of Nonmetastatic Renal Cell Carcinoma

**FIGURE 1:**
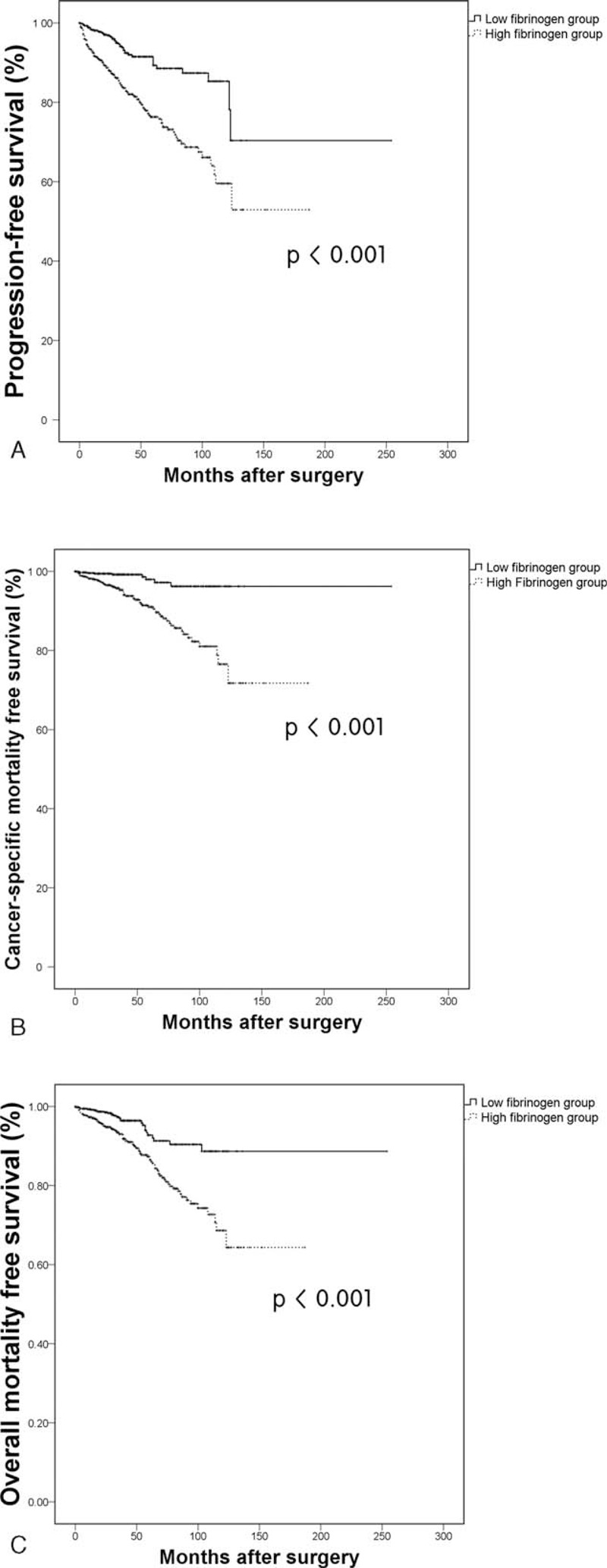
Kaplan–Meier curves for progression-free (A), cancer-specific (B), and overall survival (C) according to plasma fibrinogen level.

Kaplan–Meier analyses showed significant differences in progression (*P* < 0.001), cancer-specific mortality (*P* < 0.001), and overall mortality-free survival (*P* < 0.001) according to fibrinogen level (Figure 1). A high preoperative fibrinogen level was revealed as significant independent predictor for inferior progression (HR 1.857; 95% CI 1.314–2.624; *P* < 0.001), cancer-specific mortality (HR 3.068; 95% CI 1.477–6.372; *P* = 0.003), and overall mortality-free survival (HR 1.647; 95% CI 1.060–2.559; *P* = 0.027) in multivariate Cox analysis (Table 3).

**TABLE 3:**
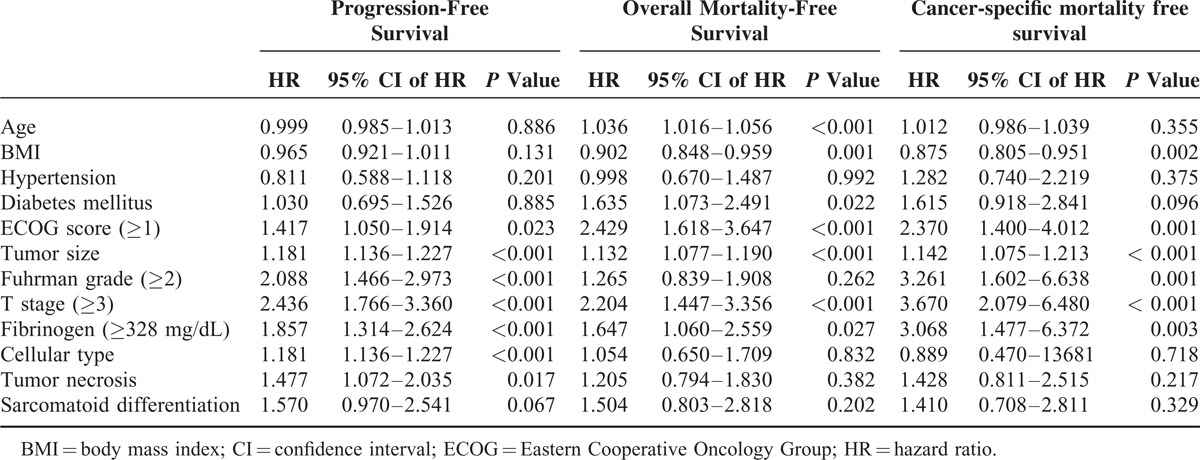
Multivariate Cox Regression Test of Possible Predictors for Progression, Overall Mortality, Cancer-Specific Mortality-Free Survival After Surgical Treatment of Nonmetastatic Renal Cell Carcinoma

Subsequently, we tried to evaluate the prognostic value of fibrinogen level separately in clear cell and nonclear cell type subgroups. When we analyzed the 1260 patients with clear cell type RCC, Kaplan–Meier analyses showed significant differences in progression (*P* < 0.001), cancer-specific mortality (*P* < 0.001), and overall mortality-free survival (*P* = 0.002) according to fibrinogen level. Multivariate Cox analysis also revealed that high preoperative fibrinogen level is an independent predictor for worse progression (HR 1.684; 95% CI 1.181–2.401; *P* = 0.004) and cancer-specific mortality-free survival (HR 3.135; 95% CI 1.436–6.845; *P* = 0.004). When we analyzed the survival outcomes in 251 patients with nonclear cell type RCC, Kaplan–Meier analysis showed significantly worse survival in terms of progression (*P* < 0.001), cancer-specific mortality (*P* = 0.027), and overall mortality-free survival (*P* = 0.002) in the high preoperative fibrinogen group. The multivariate Cox analysis also revealed that high fibrinogen level was independently related to inferior progression-free survival (HR 9.240; 95% CI 1.194–71.505; *P* = 0.033) in nonclear cell type RCC patients.

## DISCUSSION

In the present study, patients with elevated preoperative fibrinogen levels bear inferior progression, cancer-specific mortality, and overall mortality-free survival after surgical treatment of localized RCC. High fibrinogen levels were significantly associated with aggressive pathologic features such as higher Fuhrman grade and larger tumor size. Moreover, patients with high fibrinogen levels showed worse survival outcomes in progression-free survival, overall survival, and cancer-specific survival in both the clear/nonclear cell type subgroups compared with the patients with low fibrinogen levels.

Few studies have investigated the prognostic value of preoperative fibrinogen level in RCC patients; Du et al^17^ were the first, evaluating 286 patients who underwent radical nephrectomy at a single center. They observed that preoperative fibrinogen level was significantly higher in patients with a high Fuhrman grade, high T stage, and large tumor size. They also found that the preoperative fibrinogen level was an independent prognostic factor for disease-free survival and overall survival. However, their cohorts were too small and consisted of patients who underwent only radical nephrectomy, potentially resulting in selection bias. Pichler et al^18^ also retrospectively analyzed 994 patients who underwent radical or partial nephrectomy at a single tertiary center in Austria. The high preoperative fibrinogen group showed significantly worse survival outcomes in metastasis-free, cancer-specific, and overall survival (all *P* < 0.001), which corroborates our results. High fibrinogen level was significant independent predictors for worse metastasis-free survival (HR 2.47; 95% CI 1.49–4.11; *P* < 0.001), cancer-specific survival (HR 2.48; 95% CI 1.80–3.40; *P* < 0.001), and overall survival (HR 2.15; 95% CI 1.44–3.22; *P* < 0.001) in their study. But previous studies were limited by small numbers of patients and they did not weigh the histological differences between the RCC subtypes. To our best knowledge, the present study is the largest in the numbers among the previous studies about the clinical value of fibrinogen level in localized RCC. Moreover, it should be reminded that this is the first study that tried to evaluate the clinical significance of fibrinogen level in different histologic subtypes of RCC. As RCC is known to be a completely heterogeneous disease which consists of several disparate subtypes arising from different cellular origins of kidney, we believe our findings are valuable in this aspect.

The aforementioned studies tried to evaluate the clinical implications of plasma fibrinogen levels using different cut-off levels. Du et al^17^ stratified their cohorts into 3 groups (≤299, 300–399, and ≥400 mg/dL), which seem to be inferred from the upper/lower limits of the normal range of plasma fibrinogen level. Pichler et al^18^ used a different cutoff level of 466 mg/dL in their study. They stated that all possible candidates for cut-off values were calculated according to their ROC curve analyses of overall- and cancer-specific survival, and 466 mg/dL was the optimal value for discrimination of cancer-specific survival. Another report by Erdem et al^19^ recently used preoperative fibrinogen levels to accurately predict clinical outcomes after surgical treatment of RCC. They analyzed the ROC curve of fibrinogen levels for cancer-specific survival, revealing an optimal cut-off level of 343 mg/dL. In the present study, we also analyzed the ROC curve of plasma fibrinogen levels for cancer-specific survival. A cut-off level of 328 mg/dL showed the maximal Youden index (sensitivity + specificity − 1) in our ROC curve analysis, and it was determined to be the optimal cut-off value. Moreover, our cut-off level of 328 mg/dL was very similar to that of the previous study by Erdem et al.

Several possible mechanisms support the aforementioned observations. Fibrinogen can promote cell-to-cell adhesion and act like a molecular bridge between tumor cells and vascular endothelium, potentially enhancing tumor progression and metastasis.^20,21^ Moreover, elevated fibrinogen levels may protect tumor cells from the host's immune defense system. Gunji and Gorelik^22^ proposed that fibrin deposition on tumor cells may protect the tumor cell from natural killer cells’ cytotoxicity during tumor migration through the blood stream. Subsequently, Zheng et al^23^ also reported that fibrinogen could activate tumor cell adhesion with platelets, forming a dense fibrin layer around the tumor cell that can protect it from the lethal interaction with natural killer cells in the presence of thrombin. An experimental study by Palumbo et al^24^ demonstrated that fibrinogen deficiency was related to reduced incidence of spontaneous macroscopic metastases in the lungs and lymph nodes in fibrinogen-deficient mice with Lewis lung cancer. Furthermore, there were significant reductions of microscopic pulmonary metastases in the fibrinogen-deficient mice compared with primary tumor-bearing mice. They concluded that fibrinogen plays a significant role in spontaneous metastases by enhancing the adhesion and survival of metastatic emboli. Other researchers also suggested that the fibrin matrix may form stromal tissue that provides a nutrient and gas channel for malignant cells.^25,26^

Many researchers have reported a similar relationship between high plasma fibrinogen level and poor prognosis in various other malignancies. Krenn-Pilko et al^27^ retrospectively analyzed 520 patients who underwent curative treatment for breast cancer. The high fibrinogen group showed worse overall (HR 1.62; 95% CI 1.01–2.61; *P* = 0.048) and cancer-specific survival (HR 1.71, 95% CI 1.02– 2.85; *P* = 0.042) after a median follow-up time of 100 months. Seebacher et al^11^ also performed a similar retrospective study with 436 patients who underwent surgical treatment for endometrial cancer. Elevated preoperative fibrinogen level was associated with high tumor stage, unfavorable histological subtype, and advanced age in their study, and it was a significant predictor for poor recurrence-free and overall survival. Tang et al^13^ investigated 341 patients with colorectal cancer and observed that a high fibrinogen level was significantly related to advanced T stage (*P* = 0.008), venous invasion (*P* = 0.006), and a higher rate of metastases (*P* < 0.001). They concluded that patients with high fibrinogen levels showed impaired survival outcomes after surgery.

In the present study, sarcomatoid differentiation did not show any significant associations with patients’ survival in multivariate analyses using the Cox proportional hazard model. On the other hand, tumor necrosis was revealed as significant independent predictor of worse recurrence-free survival, but not in cancer-specific survival and overall survival. Considering the fact that both pathologic features have been broadly believed as adverse prognostic factors on patients’ survival, our results seem to point the opposite direction. However, several previous groups also reported nonsignificant relationship of tumor necrosis^28–31^ and sarcomatoid differentiation^31^ on patients’ survival. Moreover, Klatte et al^30^ argued that simple presence/absence analysis is not sufficient for exact evaluation of true prognosis for tumor necrosis. They recommended that more specific classification by extent of tumor necrosis is necessary for exact evaluation. Further prospective well-organized studies are needed for better understanding of these conundrums.

Our study is not free from several limitations; as a retrospective cross-sectional study, we cannot deny the possibility of inborn bias from its design. Another limitation is the relatively short follow-up of our cohorts. In addition, there are possibilities of undetected confounding factors such as systemic inflammatory conditions or cardio-pulmonary disease that may be related to the higher overall mortality observed with higher plasma fibrinogen levels. Moreover, the patients who had recurrence or progression after surgery underwent different forms of additional treatments. Those different postoperative treatments may have influenced the patients’ survival outcomes in our study.

## CONCLUSION

Increased plasma fibrinogen level was significantly associated with poor pathological outcomes and worse survival in patients with nonmetastatic RCC after surgical treatment. Patients who show high fibrinogen levels should have a more thorough follow-up protocol after surgical treatment.
